# Construction of the first genetic linkage map of Japanese gentian (Gentianaceae)

**DOI:** 10.1186/1471-2164-13-672

**Published:** 2012-11-28

**Authors:** Takashi Nakatsuka, Eri Yamada, Misa Saito, Takashi Hikage, Yuka Ushiku, Masahiro Nishihara

**Affiliations:** 1 Iwate Biotechnology Research Center, Narita 22-174-4, Kitakami, Iwate 024-0003, Japan; 2 Hachimantai City Floricultural Research and Development Center, Kamasuda 70, Hachimantai, Iwate 028-7533, Japan

**Keywords:** AFLP, Functional marker, Genetic linkage map, iPBS, Japanese gentian, marker, RAPD, REMAP, SSR

## Abstract

**Background:**

Japanese gentians (*Gentiana triflora* and *Gentiana scabra*) are amongst the most popular floricultural plants in Japan. However, genomic resources for Japanese gentians have not yet been developed, mainly because of the heterozygous genome structure conserved by outcrossing, the long juvenile period, and limited knowledge about the inheritance of important traits. In this study, we developed a genetic linkage map to improve breeding programs of Japanese gentians.

**Results:**

Enriched simple sequence repeat (SSR) libraries from a *G. triflora* double haploid line yielded almost 20,000 clones using 454 pyrosequencing technology, 6.7% of which could be used to design SSR markers. To increase the number of molecular markers, we identified three putative long terminal repeat (LTR) sequences using the recently developed inter-primer binding site (iPBS) method. We also developed retrotransposon microsatellite amplified polymorphism (REMAP) markers combining retrotransposon and inter-simple sequence repeat (ISSR) markers. In addition to SSR and REMAP markers, modified amplified fragment length polymorphism (AFLP) and random amplification polymorphic DNA (RAPD) markers were developed. Using 93 BC_1_ progeny from *G. scabra* backcrossed with a *G. triflora* double haploid line, 19 linkage groups were constructed with a total of 263 markers (97 SSR, 97 AFLP, 39 RAPD, and 30 REMAP markers). One phenotypic trait (stem color) and 10 functional markers related to genes controlling flower color, flowering time and cold tolerance were assigned to the linkage map, confirming its utility.

**Conclusions:**

This is the first reported genetic linkage map for Japanese gentians and for any species belonging to the family Gentianaceae. As demonstrated by mapping of functional markers and the stem color trait, our results will help to explain the genetic basis of agronomic important traits, and will be useful for marker-assisted selection in gentian breeding programs. Our map will also be an important resource for further genetic analyses such as mapping of quantitative trait loci and map-based cloning of genes in this species.

## Background

Gentianaceae (order Gentianales), comprises 87 genera and more than 1,500 species that are distributed worldwide. The genus *Gentiana* is the largest genus in the Gentianaceae and includes over 400 species [1]. Among them, two major floricultural species, *Gentiana triflora* and *Gentiana scabra*, the so-called Japanese gentians, have been cultivated in Japan since the 1960s. In addition to Japanese gentians, the important floricultural plant Eustoma (*Eustoma grandiflorum*), otherwise known as lisianthus, and a pharmaceutical plant, Swertia (*Swertia japonica*), also belong to the family Gentianaceae. Japanese gentians are among the most popular floricultural plants in Japan, and have unique vivid blue flowers [2]. The main Japanese gentian is *G. triflora*, which usually blooms from July to September in Japan. *G. scabra* blooms later, from September to November, and has some valuable traits, such as an open corolla and resistance to gentian brown leaf spot caused by *Mycochaetophora gentianae*[3,4]. The chromosome numbers of *G. triflora* and *G. scabra* are 2n=26, whereas those of European pharmaceutical gentians, including *Gentiana lutea* and *Gentiana punctata*, are 2n=40. It is difficult to produce interspecific hybrids among members of the genus *Gentiana* because of the different basal chromosome numbers. The nuclear DNA content in *G. triflora* is 9.11 to 9.30 pg/2C, and that in *G. scabra* is 11.35 to 11.75 pg/2C [5]. Thus, these two Japanese gentians have similar genome sizes (approximately 5×10^9^ bp/1C), which are larger than those of some other plants, e.g. *Arabidopsis thaliana* (1.5×10^8^ bp /1C) [6], *Medicago truncatula* (3.8×10^8^ bp /1C) [7], rice (4.9×10^8^ bp /1C) [8], and tomato (9.0×10^8^ bp /1C) [9].

There has been extensive molecular and physiological research on flower colors, flowering times, and cold tolerance of winter buds of Japanese gentians [10-14]. Based on these molecular studies, several genetic markers have been developed. For example, we developed genetic markers that discriminate among blue, pink, and white flower color [15,16]. To protect breeders’ rights, markers based on fragment length polymorphisms [sequence characterized amplified region (SCAR) and SSR markers] were developed to identify Japanese gentian cultivars [17,18]. The haplotypes of *W14/15* alleles, which encode an esterase involved in cold tolerance of winter buds, provided data that clarified phylogenetic relationships in the genus *Gentiana*, and allowed analysis of the pedigree and breeding history of cultivars derived from those *Gentiana* spp. [14].

Genetic linkage maps are useful for studies on genomic structure and evolution, and for identification of monogenic traits or Mendelian components of quantitative trait loci (QTLs). Therefore, they are very useful for breeding programs and are the basis for future positional gene cloning [19,20]. They are also very useful for marker-assisted breeding and introgression of beneficial traits into floricultural plants. Genetic linkage maps have been constructed for several floricultural plants, including rose (*Rosa hybrida*) [21,22], carnation (*Dianthus caryophyllus*) [23,24], Asiatic hybrid lily (*Lilium sp*.) [25], and periwinkle (*Catharanthus roseus*) [26]. However, no genetic linkage maps have been developed for the genus *Gentiana* or for any member of the family Gentianaceae, including economically important species.

The majority of floricultural plants are allogamous, but most linkage maps for crop plants have been obtained from segregating populations derived from crosses between inbred lines. Therefore, genetic linkage maps for allogamous and vegetatively reproducing species have been constructed using a double pseudo testcross strategy [25,27]. Conversely, the genetic linkage map for ryegrass (*Lolium perenne*) was constructed using a backcross population involving a double haploid (DH) tester [28,29]. Japanese gentians are also highly heterozygous plants that suffer from inbreeding depression. To obtain pure lines, the androgenic double haploid DH line, Aki6PS, was produced recently in *G. triflora* using anther culture [30].

Here, we report the construction of a genetic linkage map for Japanese gentian, *G. scabra*, using a BC_1_ population backcrossed with *G. triflora* DH line Aki6PS and 308 genetic markers. This is the first reported genetic linkage map for Japanese gentians and the first for any species belonging to the family Gentianaceae. Our results are important for understanding the genetic basis of traits and to apply molecular markers linked to mapped loci for marker-assisted selection in gentian breeding programs.

## Results

### Simple sequence repeat markers

The enriched SSR libraries from the DH line Aki6PS (*G. triflora*) yielded 19,416 clones using Roche 454 pyrosequencing. Of these, 8,932 clones (46.0%) contained SSR motifs. Only 599 clones (6.7%) were suitable for designing primer sets for SSR markers; the rest comprised reads that were too short (<200 bp) to design primers from the proximal sequences of the SSR motif. Therefore, in addition to next generation sequencing, 2,539 clones from the enriched SSR libraries were subjected to Sanger DNA sequencing analysis. As a result, 1,048 clones (41.3%) had SSR motifs within the sequence reads, and 630 clones (60.1%) could be used to design primer sets for SSR markers. These results indicated that the conventional strategy using Sanger sequencing was more efficient than next generation sequencing for developing SSR markers, without considering cost, labor, and time.

We screened 1,229 SSR markers using both parents and one F_1_ individual. Most SSR markers showed no or unstable amplification, or produced amplified fragments with unexpected lengths. Eighty SSR markers detected significant polymorphisms as co-dominant markers. In addition, 21 SSR markers were dominant markers, amplifying PCR fragments from SP6A1 and F_1_ but not from Aki6PS (Table 1). Ultimately, 101 SSR markers were available to construct the genetic linkage map. Sequence data for the markers have been deposited in GenBank/EMBL/DDBJ nucleotide sequence databases under the accession numbers (AB755678 - AB755777).

**Table 1:** List of primers used for SSR analysis

**Markers**	**Repeat motif**	**Sequence (5**^**′**^**→3**^**′**^**)**		**Product size (bp)**	**Amplification**	**Accession no.**
		**Forward**	**Reverse**			
AC1	(AC)_11_.(AC)^3^.(TA)_4_	TATCAGCAAGATGGTGCATGGTTT	CCGAAAACAGGTAAAGTGTCCATGA	160	*G. scabra*	AB755678
AC2	(AT)_3_.(AC)_11_	AGTGGGCTGGTTGGGTTTAGATTC	TACTTCAGAAGGCTGGGGTCTACG	250	*G. triflora / G. scabra*	AB755679
AC62	(AC)_6_.(CA)_8_.(CA)_7_	TAGTTTCTTTGGTGTGGGACCTGG	GGAGGAGCGTTGCTATGTTGAAGT	129	*G. triflora / G. scabra*	AB755680
CA10	(CA)_12_(TA)_3_	CTGGAAAACACCCAACACACACAT	ATCCATGTCCTCTCCGTGTAGCTC	187	*G. triflora / G. scabra*	AB755681
CA28	(CA)_9_	GCAAAATCTTTTTATTTCTTGTGTGCG	CAGCACTGTGCAAGTCATATCTTGTG	134	*G. triflora / G. scabra*	AB755682
CA36	(CA)_3_.(CA)_8_	GTGGGGAAAACAAATAGCTCTTGAGG	TCGATCATTACCTGTTGTTTCAAGAT	131	*G. triflora / G. scabra*	AB755683
CA43	(CA)_4_(AC)_7_	ATTTCCTGAAACAATGAAGTCTTATG	CGTGAAACAAAGGGATCAAAAGTATC	200	*G. triflora / G. scabra*	AB755684
CD3	(GGA)_5_.(GA)_3_	GGATCAAAAGCGATGGATCTGTTC	ATGAAGCTCCTCACCTTGTCAACC	156	*G. scabra*	AB755685
CD12	(AGT)_3_.(AGT)_7_	CCCCACCACATGGACCATAATAAC	ATATGGAGCGGGAGGTGAAGTTTT	183	*G. triflora / G. scabra*	AB755686
CD26	(AG)_3_.(TAA)_7_(CAA)_2_	TCTCACAAGCAATGCAAGACCAAT	TGGTGTACTTAGCCCTTTCGTCGT	207	*G. triflora / G. scabra*	AB755687
CD35	(GCG)_5_.(CAG)_3_	TAGGGACGTACCCGACTCAAGAAA	GAGAGAAGTAGGAGGAAGAGCCGC	225	*G. scabra*	AB755688
CD41	(ACA)_6_	TCAACGTCCAGTCCGATAATGAGA	GAACCGCACAGTAAACGCCTAATC	240	*G. triflora / G. scabra*	AB755689
CD43	(AT)_3_.(AC)_7_(TA)_6_	TGTGCATCACTTTACTGCTTTCTCTTG	CCCAAGAATCAACTTGGCTTGAAC	244	*G. triflora / G. scabra*	AB755690
CD44	(AT)_10_	TACTTGGCGGAGAGGCTTATGAAA	ACACCACAAAAGGACAGTCGTTGA	244	*G. triflora / G. scabra*	AB755691
HC5	(CA)_10_	GCTTTAGCAACTCCCTCAATCCCT	GGGAAGTGAGTAATGTTCAGGTGTC	115	*G. triflora / G. scabra*	AB755692
HC6	(CA)_14_(TA)_3_.(TA)_3_	TCCTAACATCGGTGCGAGAATGTA	AGTTTGTTTCAGAACTTGGGGCAG	115	*G. scabra*	AB755693
HC7	(CA)_10_	ACGCTGAACCTGTGAGATTTCTTG	AACTCCCATGGACCAGACACATTT	119	*G. triflora / G. scabra*	AB755694
HC18	(AC)_5_.(AC)_4_	CAAAGCAGCAGCAAATATAGGGAA	GCTCAGCAATTGTCATTCCTGCTA	156	*G. scabra*	AB755695
HC20	(CA)_3_.(TG)_6_	TCTCGGTTGAATAGAACTCACCCC	TCGTAGACCTAGCACTTTCACGCA	158	*G. scabra*	AB755696
HC23	(GA)_14_	TAGAAGTGTGTGCAATTTGGGCAT	AAAAGCAGATAAAGGAAGAGGGGG	164	*G. triflora / G. scabra*	AB755697
HC26	(CA)_9_	AGCTTGACGTTGTCTGAGGGTTTC	GCAAAAGTTTAATCGTAAGCAGGCA	176	*G. triflora / G. scabra*	AB755698
HC29	(CA)_5_.(AC)_3_.(CA)_7_(TA)_3_	CACACGCTAACAAGCACACAGCTA	TTCCTGCTTTTGTCATCCAAAGGT	194	*G. triflora / G. scabra*	AB755699
HC41	(CA)_10_.(ACA)_6_	CGTTTCTTGAGCTTATTTTTGGCG	CATTTCAGATTTCTGCCACCCTTC	219	*G. triflora / G. scabra*	AB755700
HC48	(TA)_3_T(TA)_4_G(AT)_3_.(CA)_7_	GTGTATGGCTCGAATTGTGGACTG	AAGTGGGTCTTGGGGTGAAATACC	227	*G. scabra*	AB755701
HC49	(CA)_9_	CTTTTCCCATGCATAGAATCTCCG	GCTATGGCTGGCTAGTTTCCTTTC	229	*G. scabra*	AB755702
HC71	(CA)_3_CT(CA)_11_	TGAAGTCACAAACTGCGTGTTGAA	GTGGAACCTTTGTCTTCTTCCCCT	253	*G. triflora / G. scabra*	AB755703
HC72	(CA)_10_	AGGAATCATTTGAAGGGGTTGGTT	CCGGTAATTCCCTTGAGGCATAAT	256	*G. scabra*	AB755704
HC90	(CT)_3_.(AC)_3_(CA)_12_(TA)_8_	GGTAATGCTTATGCAAGTTTGAAGG	TATTCTCCGTGTGTCCAGCTATGG	285	*G. triflora / G. scabra*	AB755705
HC91	(CA)_7_.(CA)_6_.(CA)_6_.(CA)_9_.(CT)_6_.(CT)_3_	GCATTATTTTTCTGGAGCTAGGGGA	GGGCAGCCCTGGATAATACCTCTA	286	*G. triflora / G. scabra*	AB755706
HC100	(TA)_5_.(CA)_8_.(GA)_3_	GCCGTTTTTCCTGTCCTAAGGAGT	GGGCTTGGCGAGGTAGAACTAAAA	294	*G. scabra*	AB755707
HC103	(AG)_18_	ATTTCCTCCTGCTACATGAACCCA	GCTGAGAAAGAAGCCAACTCCAAA	299	*G. triflora / G. scabra*	AB755708
HG13	(CA)_12_	CAGTTCCACTGAAATTCCGCAGAT	TATTCGTTGAAAATGGCACCCTTC	102	*G. scabra*	AB755709
HG16	(TC)_3_.(CA)_15_(TA)_3_	CCATCAAATACCCATTACCACAGAA	TCTTATCTGAACGGGAAGCCCATA	259	*G. triflora / G. scabra*	AB755710
HG26	(AC)_20_(AT)_11_	GCAATTTCTTGAACCCGTAAACCA	TGTGGAAACACGTGATGCTGAATA	149	*G. scabra*	AB755711
HG42	(CA)_15_	GCATCATATCCCAAGGTCCATCTC	GCGTAGATACCATCTCCGGTCAAC	236	*G. triflora / G. scabra*	AB755712
RC13	(CA)_14_(TA)_9_.(AC)_4_.(TA)_7_	ACGGATACGGGAGTGTATTTGTAA	TTTATTTTAATCTTGCCCCGGACC	185	*G. scabra*	AB755713
RC25	(CA)_11_	ACCAAATAGGTGTATCCGTCCGTG	TCAATCCAGGTAAATGAGACTGCTTTC	142	*G. scabra*	AB755714
ReSN7	(TA)_3_.(CA)_16_(TA)_4_C(TA)_4_	CACTGCCGGTAAGATTGGATACCT	TATAAGGACTCAGTGTCGGGACGG	173	*G. triflora / G. scabra*	AB755715
ReSN22	(CA)_13_(TA)_4_.(CA)_4_	TGTCAGTCGGGTAAGCACTTTCAG	ATCAAAGCACTTGTCTGTCCTCCC	146	*G. triflora / G. scabra*	AB755716
ReSN84	(AC)_11_	TGTAAAGGGCAAAGGAGGAAAACA	AGCTGTAAGTTGAAAACGCCTTGA	110	*G. triflora / G. scabra*	AB755717
SA3	(CA)_12_	GTACCTCTGCACATTTGTTTGGTA	GCTCTTTTCTAGCCGTTTTATGTC	182	*G. triflora / G. scabra*	AB755718
SA4	(GA)_8_	CTTCTTCTCATTCCAGTTTCACCT	AGCACCTAGATTACACGTACACCA	254	*G. triflora / G. scabra*	AB755718
SA12	(CA)_10_	GTAGGACTCTGCACAAGAAGTAGGT	ACAATAAATATGGAGGCTGTAGGC	224	*G. triflora / G. scabra*	AB755719
SH9	(AG)_15_	CTAATGTAGGACTGAATGACCCG	GCAAGAAGTAAAACGTAAAGCCTG	200	*G. triflora / G. scabra*	AB755720
SH14	(AG)_13_	AGCACATTTCCCGCTACAAC	TCTTCAGGAGGAGGAGTTTGAGT	247	*G. triflora / G. scabra*	AB755721
SN7	(CA)_8_	AAGAAACTTGGGATGTTTCAGC	AAGCTGCATTTGTTGAAGATCA	197	*G. triflora / G. scabra*	AB755722
SN24	(CA)_11_	TGGCTTGTTTTGTATTGGAGTG	GTTCATTCCCCGGATAAGAAAT	157	*G. triflora / G. scabra*	AB755723
SN27	(AC)_11_	CATGGTGAGTTGGTTGAGAAAG	CCATGAATTCAGGTGAAAGTGA	167	*G. triflora / G. scabra*	AB755724
SN28	(CACC)_7_	CAGCAGACAATCAAAACTTTCAA	TAGTCTGTTCTGTGGGGAGTGA	166	*G. triflora / G. scabra*	AB755725
SN39	(AC)_11_	ATTCTGCTCTAGCCTTCCCTCT	GAACCACATGTCCTTCATCACT	152	*G. triflora / G. scabra*	AB755726
SN40	(CA)_6_	CCAAATGGAAAATATACAGCCA	GTTCTGTTTAGGGCTTGGGGT	152	*G. triflora / G. scabra*	AB755727
SN48	(AC)_7_	TGCTGTAATTTGTGTGGCATTT	AATTTAAAAGCCAGCCTTAGCC	224	*G. triflora / G. scabra*	AB755728
SN55	(AC)_8_	AGTTAAACCTGCAAAAATTGGG	GTTTTTGTTTATACGCGCACAC	161	*G. triflora / G. scabra*	AB755729
SN73	(CA)_12_	ACAAGAAGCCAGGTTCCACTTA	TCCGATTTGAGTTGTATTGCAT	167	*G. triflora / G. scabra*	AB755730
SN87	(CA)_11_	GCACACACACCACTCCATCTTA	TGGATCAAGTTCATTGTTGCAT	175	*G. triflora / G. scabra*	AB755731
SN124	(AG)_12_	ATGTAGGACTGAATGACCCGAC	AGATGCAAAGCTTGTGGTTATT	154	*G. triflora / G. scabra*	AB755732
SN143	(AC)_10_	CCAGAAAGGGTTTTAAATGCAA	TCCTTTCAATTGCTTGAGTTTTT	151	*G. triflora / G. scabra*	AB755733
SN157	(AC)_11_	CTCACCATCACTCGATACTGGA	ACGGAAGTTTACGGTGATTCTG	171	*G. triflora / G. scabra*	AB755734
SN175	(CA)_8_	CACCAACACAAAGTTAGCTCCA	ACAGGCTTATGGGCTGTATGAG	162	*G. triflora / G. scabra*	AB755735
SN181	(CA)_6_	TTCAGTTCCACCCACACTACAC	ACACATGGTAGCTTCCCTTGAA	204	*G. triflora / G. scabra*	AB755736
SN182	(AG)_13_	AATAGAGGCGTCTTCATCATCA	CGTAACGATAAATCCCGCTAAA	151	*G. triflora / G. scabra*	AB755737
SN186	(CA)_6_	TGGTCATTTTGTGGAGTTTCAG	TGGCAGAAGAGGTGTAGAACAA	204	*G. triflora / G. scabra*	AB755738
SN224	(AC)_8_	TATGCCTTTCGTGCTCTGACTA	GGTGATAAGCTCTTATGTTGCG	151	*G. triflora / G. scabra*	AB755739
SN238	(AC)_12_	AAAAGACCAAACTTTCCCTGAC	GCTAAGCGAGCCTAAGATGTTC	154	*G. triflora / G. scabra*	AB755740
SN260	(CA)_8_(TA)_5_	CACCATAACTCGAATGTCACTG	AGCGGGCGTTACAATTAATATC	241	*G. triflora / G. scabra*	AB755741
SN282	(AC)_6_	TGGCAGAAGAGGTGTAGAACAA	TGGTCATTTTGTGGAGTTTCAG	205	*G. triflora / G. scabra*	AB755742
SN285	(AC)_12_	CCACTGATTGATGCATTTCATA	CGTTATTCTGTGCTTGAACCTG	157	*G. triflora / G. scabra*	AB755743
SN327	(AC)_11_	CAGGAGTACAACTGAGGCAGAG	ACCCAACTTCTCTCAAACGAAA	232	*G. triflora / G. scabra*	AB755744
SN328	(CA)_12_	AATTCTCCTCTTCTGCTTTTGC	TCAATTCATTCATTCAGAACCG	169	*G. triflora / G. scabra*	AB755745
SN336	(AC)_13_(AT)_9_	AAAAGAAAAAGGATGTTTGAGCA	GCAAGAAACACAACACAAGCAT	193	*G. triflora / G. scabra*	AB755746
SN343	(CA)_12_	TGGTGCAACAGGTAGATAACACA	GTTGCGTACTGACTCCTGTCTG	189	*G. triflora / G. scabra*	AB755747
SN345	(AC)_7_	ACTAGACCCTCACCAAATGCC	TGTCCCTATTCTGTTTATGCTCTT	181	*G. triflora / G. scabra*	AB755748
SN355	(AC)_8_	GACTCCATATTGCACTGGACAA	TCCCCCTCTGTACACATATCCT	214	*G. triflora / G. scabra*	AB755749
SN381	(AC)_6_	GCAAAAGAAAGCTCAAACGAAT	CACAATCTCTTGGTCCCTTTGT	172	*G. triflora / G. scabra*	AB755750
SN388	(CA)_11_	CTGCACAAATAGCAAAAGCATC	TACCCGACGATGGTAATTGTAA	167	*G. triflora / G. scabra*	AB755751
SN463	(AC)_8_	GGAAGAACTGAAGCTTCTGGAG	CAAGGAGACAACACTTGACACAG	164	*G. triflora / G. scabra*	AB755752
SN527	(TC)_4_(AC)_8_	AGCTCCCAAAGTATCACTCCAA	CTGACATTTTACCAAGCCACAA	185	*G. triflora / G. scabra*	AB755753
SN563	(AT)_7_	CATGTCCTCTGTGAGGTCAAAA	GTCGGATTTTGGAGATTCGTAG	158	*G. triflora / G. scabra*	AB755754
SN578	(CA)_8_	TCCCCCTCTGTACACATATCCT	GACTCCATATTGCACTGGACAA	214	*G. triflora / G. scabra*	AB755755
SN583	(CA)_10_	TTTCATTGCACCTGGACATAAG	AAAGTCAATGAATTCCAAAGGC	151	*G. triflora / G. scabra*	AB755756
SP1	(CA)_13_	ATGATGATTGGTCAGCCTCGG	TGCTGGTTTGGTATTGAACTCTTCG	216	*G. triflora / G. scabra*	AB755757
SP8	(AC)_12_	CTCTGCAACCAAACAATACCCCAT	GGAAGGGGATGAATCGAGGAGTTA	327	*G. triflora / G. scabra*	AB755758
SP15	(GA)_17_	GGATTTCGAGCGATTTTCTACTC	GCAGAAGTTTTCCTTACGATGG	248	*G. triflora / G. scabra*	AB755759
SP17	(AC)_7_	AAAACTCAGGAAAGCCGCAATA	AATAGCCTCCCAAGCACCCTATAA	181	*G. triflora / G. scabra*	AB755760
SP21	(AC)_13_	GGGACCAACACCATTATTTCCCTT	GGAAGATCACATTTATGCCCCTCA	307	*G. triflora / G. scabra*	AB755761
SP28	(TG)_12_.(GA)_26_	CCGCGTAAGTGGCATACTCATAA	GTTACTTCCTTCAACCAGGGTATCTG	334	*G. triflora / G. scabra*	AB755762
SP29	(AC)_10_	TGTAGTTGAGCAAGCCAAATCTGC	GTGTAAGCCCAATAAGTCAATTCATGC	212	*G. triflora / G. scabra*	AB755763
SP37	(AC)_11_(CA)_6_	CAGAAGGCTGGGGTCTACGAAATA	CCTAGTGGGCTGGTTGGGTTTAG	250	*G. triflora / scabra*	AB755764
SP43	(AC)_15_	ACTTGGGGCAGCAGAAACTTAACA	TTGACCTAACATCGGTGCGAGAAT	107	*G. scabra*	AB755765
SP44	(CA)_8_.(TA)_11_(CA)_12_	CGAATAAAAGGAACCGAACCCAAT	AAGGGACGCCGCTACTTTCTACAG	281	*G. scabra*	AB755766
SP47	(CA)_11_	GAAAGTTGAATTACTTGCAGGCTC	TTCATATTAGACGGGTTTGGGTC	280	*G. scabra*	AB755767
SP48	(AC)_10_	CCGATTCAACGCCTAGCAACT	TCAAGAAACACGATACTGATGTGGG	271	*G. triflora / G. scabra*	AB755768
SP49	(AC)_10_(AT)_6_	TCCGTTGTTCTTCGTAAAGGTTGG	AGAGTGATTTCTTCTGGCTCGTTTCTT	337	*G. triflora / G. scabra*	AB755769
SP52	(AC)_14_	CTGAATACAAGGTTTAGCTCCTTCA	ATTAGCTCGGTTTTGCACTTAGAC	291	*G. scabra*	AB755770
SP53	(AT)_6_	GCCGTATCCAACAAAAGAAACA	TTACATTCCTCCCACTTAAAAGCC	322	*G. scabra*	AB755771
SP57	(TA)_12_(GA)_19_	CGTGTAATTCGGTTAAATCCCTTCC	AAAAGTGGTTATTCGGTTCGGGTT	231	*G. scabra*	AB755772
SP58	(AG)_17_	GATGCTAGATGGGCTAGAGGAAAGA	CAGTGCAAAGCAACTCGATAAGGT	129	*G. scabra*	AB755773
SP61	(CA)_13_(TA)_9_	ACCCTCACTAAACCTTCACAGCGT	CACAGAGCATATTACCGCTTCTTGA	226	*G. scabra*	AB755774
SP63	(AC)_7_	AGGAACAATCTTGACTAGCCTCGG	GAAGTGAGAGTTGGATTAGGGTGAAAA	264	*G. triflora / G. scabra*	AB755775
Tdssr3	(AG)_17_	TCAAAAGCTCAGTAACTAAACCTTCA	CCCAATAACAGTATAAGGACCAATCT	271	*G. triflora / G. scabra*	AB755776
Tssr3	(CAA)_7_	CATGTTGATAAAGACTGGAAAGAATG	CCAAAACTAGTCCAGGTAAAATTCTC	154	*G. triflora / G. scabra*	AB755777

### Retrotransposon microsatellite amplified polymorphism markers

**Figure 1:**
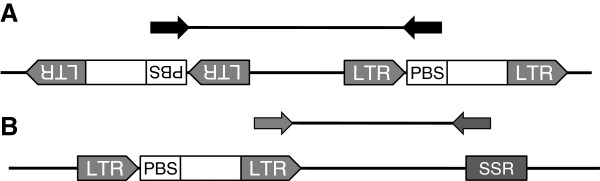
**Schemes of iPBS and REMAP. A**) iPBS is an efficient method which isolates long terminal repeat (LTR) sequences of retrotransposons [32]. Most fragments that are amplified between two retrotransposons by iPBS primers annealed at primer binding sites (PBS) contain putative LTR sequences. **B**) Retrotransposon and microsatellite amplified polymorphism (REMAP) amplified DNA fragments located between LTRs proximal to simple sequence repeats (SSRs).

To increase the number of available genetic markers for Japanese gentian, we developed some retrotransposon microsatellite amplified polymorphism (REMAP markers) using a retrotransposon. A retrotransposable element *GsTRIM1* (terminal repeat retrotransposon in miniature) has been reported in *G. scabra*[31]. Therefore, to isolate novel LTRs of retrotransposons from Japanese gentians, we used iPBS technology as described by Kalendar et al. [32] (Figure 1A). Genomic DNA was amplified using eight iPBS primers (shown in Table 2), and then subjected to sequencing analysis. Three putative LTR sequences, 758 bp (88% identity), 735 bp (86%) and 318 bp (91%), were found in the comparative sequence analyses among 64 independent clones. Although eight primers could be designed from the three putative LTR sequences, a few amplified fragments were obtained using each LTR primer during inter-retrotransposon amplified polymorphism (IRAP) analysis (data not shown). Therefore, each LTR primer was explored in combination with ISSR primers to create REMAP markers (Figure 1B). ISSR primer sets provided from the University of British Columbia (set #9) were pre-screened using Aki6PS genomic DNA. Seventeen primers amplified more than eight stable fragments (data not shown). Seven primer sets combining LTR and ISSR, as shown in Table 3, produced reproducible amplified fragments. These primer combinations provided stable and reproducible results at an annealing temperature of 60°C. REMAP markers were more efficient and reproducible than IRAP and ISSR markers in Japanese gentians.

**Table 2:** List of primers used for iPBS amplification

**Primer**	**Sequence (5**^**′**^**→3**^**′**^**)**	**Annealing**
iPBS2076	GCTCCGATGCCA	54°C
iPBS2077	CTCACGATGCCA	54°C
iPBS2079	AGGTGGGCGCCA	60°C
iPBS2080	CAGACGGCGCCA	63°C
iPBS2083	CTTCTAGCGCCA	52°C
iPBS2085	ATGCCGATACCA	52°C
iPBS2374	CCCAGCAAACCA	52°C
iPBS2378	GGTCCTCATCCA	51°C

**Table 3:** List of primers used for REMAP analysis

**Primer**	**Sequence (5**^**′**^**→3**^**′**^**)**	
	**LTR**	**ISSR**
REMAP1	GTAGGCACTTCAAGAATTCCACTT	(AC)_9_T
REMAP2	GTAGGCACTTCAAGAATTCCACTT	(CAC)_7_G
REMAP3	GTAGGCACTTCAAGAATTCCACTT	(ACC)_6_G
REMAP4	CTAAGGGAAGCTCATAAGTTTAGGC	(AC)_9_C
REMAP5	TACCCCTTGCCCAAGTTATCCTGT	(AG)_9_C
REMAP6	TAGTACAACGGTAAGCGCTTGATC	(AC)_9_G
REMAP7	TAGTACAACGGTAAGCGCTTGATC	(ACC)_6_T

### Amplified fragment length polymorphism markers

Our preliminary experiments showed that conventional amplified fragment length polymorphism (AFLP) amplification using combinations of selective primers with three selective nucleotides resulted in too many faint and overlapping fragments in gentian (data not shown). To solve this problem, we added a fourth selective nucleotide to the *Mse* I primer, as described by Remington et al. [33] (Table 4). AFLP and RAPD markers are dominantly inherited; therefore, we identified only *G. scabra* SP6A1-specific fragments among many amplified fragments because Aki6PS-specific fragments were not segregated in the BC_1_ population backcrossed with *G. triflora* Aki6PS. Fourteen primer sets, which produced more than 10 SP6A1-specific fragments per primer combination, were selected to generate the genetic linkage map (Table 4).

**Table 4:** List of primers used for AFLP analysis

**Marker**	**Sequence of selective primers (5**^**′**^**→3**^**′**^**)**	
	***Mse *I**	***Eco *RI**
Pre-selective	GATGAGTCCTGAGTAAC	GACTGCGTACCAATTCA
M01E1	GATGAGTCCTGAGTAACACA	NED-GACTGCGTACCAATTCAAC
M01E8	GATGAGTCCTGAGTAACACA	JOE-GACTGCGTACCAATTCAGG
M02E1	GATGAGTCCTGAGTAACACT	NED-GACTGCGTACCAATTCAAC
M02E3	GATGAGTCCTGAGTAACACT	FAM-GACTGCGTACCAATTCACA
M03E1	GATGAGTCCTGAGTAACACC	NED-GACTGCGTACCAATTCAAC
M03E2	GATGAGTCCTGAGTAACACC	JOE-GACTGCGTACCAATTCAAG
M05E8	GATGAGTCCTGAGTAACATA	JOE-GACTGCGTACCAATTCAGG
M06E1	GATGAGTCCTGAGTAACATT	NED-GACTGCGTACCAATTCAAC
M07E2	GATGAGTCCTGAGTAACATC	JOE-GACTGCGTACCAATTCAAG
M08E1	GATGAGTCCTGAGTAACATG	NED-GACTGCGTACCAATTCAAC
M08E3	GATGAGTCCTGAGTAACATG	FAM-GACTGCGTACCAATTCACA
M13E3	GATGAGTCCTGAGTAACTTA	FAM-GACTGCGTACCAATTCACA
M23E3	GATGAGTCCTGAGTAACTAC	FAM-GACTGCGTACCAATTCACA
M24E3	GATGAGTCCTGAGTAACTAG	FAM-GACTGCGTACCAATTCACA

### Long random amplification polymorphic DNA markers

We first screened for reproducible and stable random amplification polymorphic DNA (RAPD) markers using 47 primers, as described by Debener and Mattiesch [21] and by Yamagishi et al. [34]. Among them, twelve 15-mer primers (Table 5) amplified bands reproducibly, and were selected for genetic analysis of Japanese gentians. An average of 10 fragments (< 3 kb in length) with two to seven SP6A1-specific fragments were amplified per primer.

**Table 5:** List of primers used for long RAPD analysis

**Primer**	**Sequence (5**^**′**^**→3**^**′**^**)**
P474	AGGGCCATTGCACCG
P615	GCCGTGGACTGCAGA
P616	GTATGAACGGTGACC
P618	TCAGGTTATCGCCCC
P620	GGCTATTCAGCTGGC
P622	GCGATGACACAGGAC
P630	CCTGCAGCTCACGGA
P642	GGACCACCGTAAGCC
P646	CACCCGTAGCGTGAG
P651	CTCCCGGCGAGTGGA
P652	CTTCGCTCGAACGCG
P654	TGATAGCGCCACCCG

### Functional markers

In Japanese gentians, there has been extensive physiological and molecular research on flower pigmentation, flowering time, and cold tolerance of winter buds, and the nucleotide sequences of some important genes have already been deposited in public databases. In addition, our previous study reported significant insertions/deletions (in/dels) in introns and untranslated regions, representing molecular polymorphisms that could be used to discriminate among cultivars and species of Japanese gentians [17]. Therefore, we first identified the intron and proximal sequences of chalcone synthase (*CHS*) and the flowering locus T1 (*FT1*) and *FT2* genes from both *G. triflora* Aki6PS and *G. scabra* SP6A1. Comparative sequencing analysis identified significant in/del polymorphisms with the intron regions of *CHS* and *FT1,* and marker primer sets were designed in the proximity of the in/dels of each gene (Table 6). For chalcone isomerase (*CHI*), flavanone 3-hydroxylase (*FHT*), flavonoid 3^′^,5^′^-hydroxylase (*F3*^*′*^*5*^*′*^*H*), anthocyanidin synthase (*ANS*), *MYB3*, basic helix loop helix 1 (*GtbHLH1*), terminal flowering 1 (*TFL1*) and *W14/15* genes, we used the primer sets reported in previous studies [11,12,14,16,17]. No in/dels were detected in the genomic sequences of *FT2* and *TFL1* genes, but some SNPs were detected using restriction enzymes. Therefore, *FT2* and *TFL1* markers were distinguished between Aki6PS and SP6A1 as cleaved amplified polymorphic sequence (CAPS) markers, when their amplified fragments were digested by *Pst* I and *Sau*3A I, respectively.

**Table 6:** List of primers used for functional markers

		**Sequence (5**^**′**^**→3**^**′**^**)**	
		**Forward**	**Reverse**
**Anthocyanin biosynthesis**			
*CHS*	Chalcone synthase	TGTGCAAAGTTGATTTTATTCGAC	ATGTCAGAAAGGAGGGTCCAATGG
*CHI*	Chalcone isomerase	GAGTGGTTAAGCAGATGACACGAC	AAGAAAATTGACAACATGCAGAAG
*FHT*	Flavanone 3-hydroxylase	TTGCACCTGAAGTAGAATTTTACA	TTCTGACAGAACTTCAAGCAATTT
*F3*^′^*5*^′^*H1*	Flavonoid 3^′^,5^′^-hydroxylase	TCCATTGATTAAAATGAGGGACCA	TATGGTAAGTTGGGGATGTCTGAT
*ANS*	Anthocyanidin synthase	ATGTCAACTTTTTATTGGTCCTAA	GAGCACAGCAAGAACTTTGGTAGC
*MYB3*	Transcription factor	CAATGCAGCAACATTTACACTACTCCCA	AAGAATCCATGAATGTAGCAGCAGCATC
*bHLH1*	Transcription factor	AAGGTGATCGTTGTGAAAATGTCT	GGCCGTCTAGTTTGGTGGTTGGTT
**Flowering time**			
*FT1*	Flowering locus T	CCTCAGGGAATACCTGCACTGTTT	ATCCCTCTAAAGTTTAGGTGTGTTATAAG
*FT2*	Flowering locus T	TCTTTGACTCTCTTGCTTTCTTGATGA	ACCATCTTTCTACGACCGTTGCAT
*TFL1*	Terminal flowering 1	AAGATTATGGATTTGTACTCTTAGTCT	TAACTAATGATAAGATTATGTGAAG
*W14/15*	Esterase	CTAGTCTCTACCATTTGTCCC	TCTACAAACAATGCACCTGG

### Segregation of stem pigmentation trait

The *G. triflora* breeding line Aki6PS has a green stem color, while stems of the *G. scabra* breeding line SP6A1 and F_1_ are red because of the accumulation of anthocyanins. Among BC_1_ progeny, 44 and 49 individuals showed green- and red-stem color phenotypes, respectively. Segregation of stem color fitted a 1:1 ratio based on a chi-square test at *P* = 0.05, suggesting monogenetic inheritance of this trait.

### Genetic map construction

**Figure 2:**
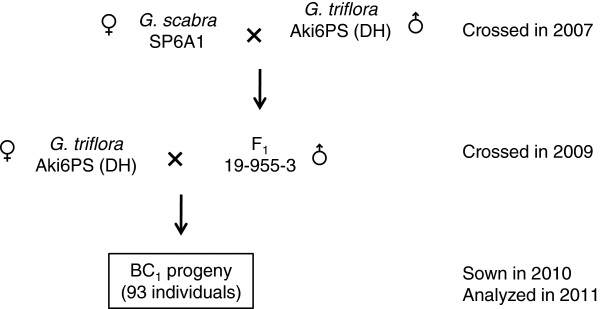
**Production of BC**_**1 **_**progeny for genetic linkage analysis. ***G. scabra *breeding line SP6A1 is an unfixed breeding line, because most Japanese gentians exhibit inbreeding depression. Aki6PS is a double haploid line (DH) generated from *G. triflora *cv. ‘Ashiro-no-Aki’ using anther culture. Crossing the DH line Aki6PS back into one F_1 _individual yielded 93 BC_1 _progeny.

**Figure 3:**
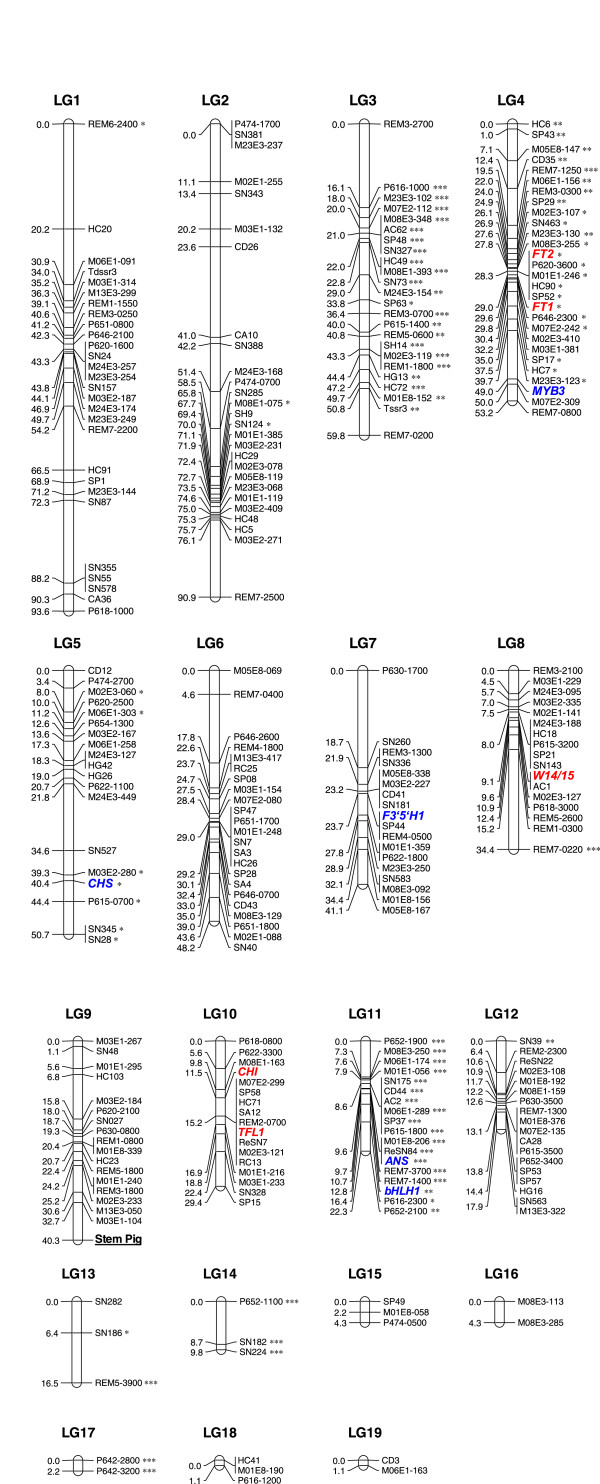
**Genetic linkage map of *G. scabra. ***Genetic linkage map of *G. scabra. *based on a BC_1 _mapping population generated by crossing *G. scabra *SP6A1 × *G. triflora *Aki6PS DH line. Map was generated with 307 (101 SSR, 103 AFLP, 54 RAPD, 38 REMAP and 11 functional) polymorphic markers using JoinMap version 4.1 at an LOD value of 9.0, with the Kosambi mapping function. In total, 273 markers (97 SSR, 97 AFLP, 39 RAPD, 30 REMAP and 10 functional) were mapped on 19 linkage groups (LG), which spanned 613.7 cM. Distortions at the 5%, 1% and 0.1% level are indicated as *, ** and ***, respectively. AFLP, RAPD, and REMAP markers are indicated as M*black circle*E*black circle*-, P*black circleblack circle*- and REM*black circle*-, respectively. Of the 10 functional markers, flowering related genes, *FT1*, *FT2*, *TFL1 and W14/15* are shown in red, and flower pigmentation related genes *CHS*, *CHI*, *ANS*, *F3*′*5*′*H1, MYB3* and *bHLH1* are shown in blue. Stem pigmentation (Stem Pig), which represents a phenotypic trait, is underlined.

We used a population of 93 BC_1_ from *G. scabra* SP6A1 backcrossed with *G. triflora* Aki6PS DH line (Figure 2). In total, 308 markers, comprising 101 SSR markers, 103 AFLP markers, 54 RAPD markers, 38 REMAP markers and 11 functional markers, and one phenotypic marker were grouped with a LOD score of 9.0. As a result, 19 linkage groups containing at least two markers could be assembled with 273 markers (Figure 3). Thirty-five markers were unmapped and ungrouped in this linkage map, and more RAPD (27.8%) and REMAP (21.0%) markers were excluded from the linkage groups, compared with SSR (3.9%), AFLP (5.8%) and functional markers (9.0%). Of the 10 functional markers, *FT1* was closely linked to *FT2,* an ortholog of *FT1*. Stem color locus as a phenotypic trait was closely linked to the M03E1-104 AFLP marker in LG9. In total, 19 linkage groups were identified, covering 613.7 cM with an average distance of 2.2 cM between each pair of markers. The size of the linkage groups ranged from 1.1 to 93.6 cM. Twenty-seven, 16, and 38 markers showed distorted segregation at the 5%, 1%, and 0.1% levels, respectively, based on the chi-square test. Five linkage groups (LG3, LG4, LG11, LG14, and LG17) were assembled from markers with distorted segregation at the 0.1% level in the genetic map of Japanese gentian.

## Discussion

We used 454 FLX Titanium pyrosequencing of an SSR-enriched library to develop SSR markers from Japanese gentians. Recently, next generation sequencing has been used successfully to develop several genetic markers, including SSRs [35-37]. In our study, out of approximately 20,000 sequences from the enriched SSR library, only 3.0% could be used to design primers suitable for SSR markers. In contrast, 24.8% of the sequences derived from conventional Sanger DNA sequencing were suitable for designing primers for SSR markers. This is because the reads obtained from pyrosequencing in this study are shorter and less accurate than those obtained from Sanger sequencing. Similarly, in the honey bee (*Apis mellira*) and zebrafish (*Danio rerio*), only 1.2% to 5.4% sequences obtained by 454 FLX Titanium pyrosequencing could be used to design primers for SSR markers [37]. In spite of its flaws, next generation sequencing is cheaper and less labor-intensive than conventional Sanger DNA sequencing. As next generation sequencing technology develops further, for example, the long-read protocols based on the GS FLX+ system, this technology will become more efficient for development of SSR markers.

Retrotransposons can be used as molecular markers because their integration creates new junctions between genomic DNA and their conserved ends [38]. IRAP and REMAP are simple methods that do not require restriction enzyme digestion or ligation to generate marker bands [38,39]. However, the limiting factor for developing genetic markers based on LTR retrotransposons for new plant species is the availability or otherwise of retrotransposon sequences. In Japanese gentian, three dispersed transposable elements, *GsTRIM1, Tgt1* (*gypsy-Ty3* retrotransposon), and *GtMITE1*, have been identified [31,40]; however, there are low copy numbers of *Tgt1* and *GtMITE1* in the gentian genome [40]. We used iPBS amplification [32], which is based on the universal presence of primer binding sites (PBS) of LTR retrotransposons, to identify new LTR sequences from Japanese gentian. Three putative LTR sequences were predicted from overlapping regions among independent sequences obtained by iPBS amplification. These sequences did not correspond to the terminal direct repeat (TDR) of *GsTRIM1* and LTR of *Tgt1*. We designed primers from these newly identified LTR sequences (Table 3). However, no or few fragments were amplified by the putative LTR primers from any Japanese gentian lines, probably because the LTR retrotransposons were too far apart for efficient amplification. Therefore, these LTR primers were not suitable as IRAP markers. Conversely, REMAP primers yielded more reproducible and stable fragments in Japanese gentians than IRAP and ISSR, and were used to generate the genetic linkage map.

RAPD and AFLP technologies are conventional molecular markers. Their advantages are that they do not require sequence information and have a relatively low start-up cost [41-43]. However, certain procedures must be modified to use RAPD and AFLP markers for Japanese gentians, probably because the large genome size and heterogeneity. RAPDs using10-mer primers and AFLP using three selective nucleotides resulted in too many faint and overlapping fragments (data not shown), whereas long-RAPDs using 15-mer primers amplified reproducible and stable fragments at higher annealing temperatures. The use of 15-mer primers also led to more effective amplification of polymorphic fragments than 10-mer primers in grape [43], rose [21], and Asiatic hybrid lily [34]. The AFLP markers produced reproducible fragments using *Mse* I-selective primers with four selective nucleotides in Japanese gentians. In conifer, which has a very large genome (2×10^10^ bp), the use of selective primers with a fourth selective nucleotide also improved amplification of reproducible fragments [33]. Therefore, markers developed by modified RAPD and AFLP were used to generate the gentian genetic linkage map (see below).

A genetic linkage map with 273 markers was constructed using an interspecific BC_1_ population. Nineteen linkage groups were obtained for the BC_1_ population at an LOD value of 9.0, and encompassed 613.7 cM (Figure 3). Analysis of published saturated maps for tomato (n = 12, 1,283 cM) [44], melon (n = 12, 1,150 cM) [45], and lettuce (n = 9, 1,505 cM) [46] using the JoinMap program showed that an average map distance of 100 to 150 cM can be expected per chromosome regardless of its physical length. We estimate the map length of Japanese gentians (n =13) to be 1,300 to 1,950 cM. The present genetic linkage map probably covers less than half of the genome; therefore, more genetic markers are required to saturate the linkage map of Japanese gentians.

In the BC_1_ progeny of Japanese gentians, 30.0% of markers showed segregation distortion. These skewed segregations were also reported in other ornamental plants species, such as the diploid rose [21] and Asiatic hybrid lily [25]. The causes of segregation distortion are not well understood. Segregation distortion has been reported in *Rhododendron*[47], Asiatic lily [25] and carnation [23,24], and it was assumed to represent hybrid sterility genes and gametophytic selection genes in interspecific crossing. The BC_1_ population used in this study was derived from interspecific crossing between *G. triflora* and *G. scabra,* and the reciprocal cross showed hybrid weakness. Therefore, hybrid sterility and gametophytic selection genes might be responsible for the segregation distortion in Japanese gentian populations.

Functional markers are those derived from polymorphic sites within genes responsible for phenotype traits [48]. Ten functional markers for genes involved in important traits, such as flower pigmentation (*CHS*, *CHI*, *F3*^*′*^*5*^*′*^*H*, *ANS*, *MYB3* and *bHLH1*), flowering time (*FT1*, *FT2* and *TFL1*) and cold tolerance of winter buds (*W14/15*), were mapped to the 19 linkage groups of Japanese gentian (Figure 3). Our previous research revealed that white-flowered gentians resulted from the functional deficiency of *ANS, a* structural enzyme*,* or *MYB3*, an anthocyanin biosynthetic transcription factor. Genetic analysis showed that the *ANS* locus was not linked to the *MYB3* locus [11,49]. Consistent with these observations, *ANS* and *MYB3* were assembled into different linkage groups: LG11 and LG4, respectively (Figure 3). Interestingly, two orthologs of the flowering hormone florigen, *FT1* and *FT2*, were closely linked (LG4). These findings will be useful for determining the molecular functions of these genes and to study the genetic regulation of flowering time in gentian, although further research is necessary. The mapping of functional markers corresponding to important agricultural traits will be accelerated by further molecular and physiological studies on Japanese gentians. Our genetic linkage map will be useful for mapping QTLs associated with various traits, and for improving Japanese gentian breeding programs. The map is also applicable to other members of the Gentianaceae family, including several other economically important species.

## Conclusions

This study presents the first genetic linkage map for Japanese gentian. The map was constructed using four different types of molecular markers. It was produced from genotypes of 93 BC_1_ progeny derived from a DH line and included 274 markers (97 SSR, 97 AFLP, 39 RAPD, 30 REMAP, 10 functional markers, and 1 phenotypic trait). The map revealed 19 linkage groups that covered 613.7 cM, with an average intermarker distance of 2.2 cM. This map is a starting point for mapping single or quantitative trait loci affecting agronomically important phenotypes, and will be useful for research on gentian genetics and breeding.

## Methods

### Plant Materials

In total, 93 BC_1_ progeny were derived from the interspecific backcross Aki6PS×SP6A1 (Figure 2). Aki6PS is a double haploid (DH) line derived from *Gentiana triflora* cv. ‘Ashiro-no-Aki’ using anther culture [30]. SP6A1 is a breeding line derived from the *G. scabra.* F_1_ was produced by crossing a female Aki6PS with a male SP6A1. BC_1_ progeny were produced by crossing a female Aki6PS with pollen of a single F_1_ individual, because the reciprocal hybrid (F_1_×Aki6PS) showed hybrid weakness and it was difficult to obtain sufficient progeny.

Genomic DNA was isolated from young leaves (500 mg) of each individual using Nucleon PhytoPure (GE Healthcare, Little Chalfont, UK) and stored at −20°C until use.

### Simple sequence repeat markers

SSR markers were developed as described by Sato-Ushiku et al. [18]. Genomic DNA was isolated from either *G. triflora* Aki6PS or *G. scabra* SP6A1, and then digested by either *Alu* I, *Hae* III, *Mse* I, or *Rsa* I. The digested genomic DNA was hybridized with biotin-labeled (CA)_15_ or (GA)_15_ probes. SSR-enriched libraries were mixed and subjected to sequencing analysis using 454 GS FLX Titanium pyrosequencing (Roche, Basel, Switzerland). The SSR-enriched library was also subcloned into the pGEM-T Easy vector (Promega, Madison, WI, USA), and subjected to sequencing analysis using BigDye terminator version 1.1 cycle sequencing kit and an ABI PRISM 3130xl Genetic Analyzer (Applied Biosystems by Life Technologies, Foster City, CA, USA). SSR markers were predicted and developed using Read2Marker software with the default parameters [50].

PCR amplification was performed in a 20-μL reaction mixture containing 10 ng genomic DNA, 0.5 μM each primer (Table 1), 0.2 mM dNTPs, 1 × Ex buffer, and 0.25 units *Ex Taq* polymerase (Takara Bio, Otsu, Japan). The PCR conditions were as follows: 94°C for 2 min; 30 cycles at 95°C for 20 s, 60°C for 40 s, and 72°C for 1 min; and a final extension at 72°C for 5 min. The amplified fragments were separated on a high-efficiency genome scanning (HEGS) running system (Nihon Eido, Tokyo, Japan) [51]. In brief, 2 μL of each sample per lane was loaded onto polyacrylamide gels consisting of a stacking gel (5% [w/v] bis-polyacrylamide [29:1] containing 0.5 M Tris–HCl, pH6.8) and running gel (15% [w/v] bis- polyacrylamide [29:1] containing 1.5M Tris–HCl, pH 8.8) and electrophoresed in 1× Tris–glycine buffer (25 mM Tris–HCl, 1.92 M glycine, pH 8.3) at 300 V for 2 h. The sizes of the fragments were estimated based on a 20-bp ladder (Takara Bio). The gels were stained with SYBR Gold nucleic acid gel stain (Molecular Probes by Life Technologies, Helsinki, Finland), and photographed and analyzed using a ImageQuant LAS-4000 luminescent image analyzer (GE Healthcare).

### Retrotransposon microsatellite amplified polymorphism markers

The sequences of LTRs of retrotransposons were isolated from gentian genome using the iPBS approach [32]. PCR was performed in a 25-μl reaction mixture containing 1 μg genomic DNA, 1×*Ex* buffer, 1 μM each primer, 0.2 mM dNTPs, and 1 unit *ExTaq* DNA polymerase. The PCR program was as follows: 1 cycle at 95°C for 3 min; 30 cycles of 95°C for 15 s, 50–70°C for 1 min, and 72°C for 1 min; and a final extension step of 72°C for 5 min. The sequences and annealing temperatures of iPBS primers are shown in Table 2. The reaction mixtures were purified using a Microspin S-400 HR column (GE Healthcare), and then subcloned into the pCR4-TOPO TA cloning vector (Invitrogen in Life technologies, CA). Sixty-four independent clones were subjected to sequencing analysis, as above. Putative LTRs were found by comparative analysis among sequences obtained by the iPBS approach using stand-alone BLAST [52].

The REMAP markers were created by combining LTR primers with ISSR primers, as shown in Table 3. Each 20-μl reaction mixture comprised 20 ng genomic DNA, 1×*Ex* buffer, 0.2 mM dNTPs, 0.2 μM each primer, and 1 unit *Ex Taq* polymerase. The PCR program was as follows: 94°C for 4 min; 30 cycles of 94°C for 40 s, 60°C for 40 s, and 72°C for 2 min; followed by final extension at 72°C for 5 min. The amplified fragments were separated on 1.0 or 1.6% (v/v) agarose gels in TAE buffer, and then photographed and analyzed with an ImageQuant LAS-4000 system after staining with ethidium bromide.

### Amplified fragment length polymorphism markers

AFLP analysis was performed as described by Vos et al. [41] with some modifications. One microgram of genomic DNA from each individual was digested with two restriction enzyme, 25 units of *Mae* I and 50 units of *Eco*R I, in a reaction volume of 25 μl with restriction enzyme buffer (10 mM Tris–HCl, pH 7.5, 10 mM magnesium acetate, 50 mM potassium acetate, 5 mM dithiothreitol, 0.005%(v/v) bovine serum albumin, pH 7.5) at 37°C for 3 h. The restriction enzyme solution was added to 10 μl adapter ligation solution (1×restriction enzyme buffer, 1mM ATP, 2.5μM *Eco*R I adapter, 2.5μM *Mse* I adapter, 50 units T4 DNA ligase), and then incubated at 37°C overnight. To stop the reaction, 500 μl T_10_E_0.1_ buffer (10 mM Tris–HCl, 100 mM EDTA, pH 8.0) was added to the ligation solution.

Pre-amplification was conducted in a 25-μl reaction volume containing 2.5 μl diluted adaptor-ligated DNA, 0.4 μM EcoRI+A primer, 1.6μM MseI+C primer, 200 μM dNTPs, 1×*ExTaq* buffer, and 1.25 units *ExTaq* DNA polymerase (Takara-bio). The PCR program was as follows: 94°C for 2 min; 20 cycles of 94°C for 30 s, 56°C for 1 min and 72°C for 1 min; followed by final extension at 72°C for 10 min. The PCR products were diluted 100-fold in T_10_E_0.1_ buffer. Four labeled-*Eco*R I selective primers with three selective nucleotides (NED-AAC, JOE-AAG, FAM-ACA and JOE-AGG) and 10 *Mse* I selective primers with four selective nucleotides (CACA, CACT, CACC, CATA, CATT, CATC, CATG, CTTA, CTAC and CTAG) were used for selective amplification. We screened 14 primer combinations because they provided the highest number of heterozygous bands (Table 4). For the selective PCR reaction, the 10-μl reaction mixture consisted of 5 μl pre-selective PCR product, 1×*ExTaq* buffer, 200 μM dNTPs, 0.5 μM labeled-*Eco*R I-ANN primer, 0.5 μM *Mse* I-ANNN primer, and 0.5 units *ExTaq* DNA polymerase. The touchdown PCR profile was as follows: 1 cycle at 94°C for 30 s, 68°C for 30 s, and 72°C for 1 min; 17 cycles with the annealing temperature reduced by 0.7°C/cycle; 23 cycles with an annealing temperature of 56°C; and a final extension at 72°C for 10 min. PCR products were separated and detected using an ABI 3130 genetic analyzer. The sizes of the amplified products were calculated based on internal standard DNA (GeneScan-500ROX size standard, Applied Biosystems) using GeneMapper software (Applied Biosystems).

### Long random amplified polymorphic DNA markers

The long RAPD markers were developed as described by Debener and Mattiesch [21] and Yamagishi et al. [34]. After pre-screening, twelve 15-mer primers (Table 5) were selected to generate the genetic linkage map. The 20-μl reaction mixture comprised 20 ng genomic DNA, 1×*Ex* buffer, 0.2 mM dNTPs, 0.4 μM 15-mer primer, and 1 unit *Ex Taq* polymerase. The PCR program consisted of 94°C for 90 s; 40 cycles of 95°C for 30 s, 53°C for 1 min 30 s, and 72°C for 2 min; followed by final extension at 72°C for 5 min. The amplified fragments were separated on 1% (v/v) agarose gels in TAE buffer, and then photographed and analyzed as described in the REMAP markers section.

### Functional markers

Our previous research revealed that the intron lengths of several flavonoid biosynthetic genes, including *CHS*, *CHI*, *FHT*, *F3*^*′*^*5*^*′*^*H*, *ANS,* and the transcriptional factors *GtMYB3* and *GtbHLH1* exhibited significant polymorphisms among Japanese gentian cultivars [10,16,17]. Primers for *GtFT1*, *GtFT2,* and *GtTFL1*, which are regulator genes of flowering time [12], were also designed from the difference in the genomic sequences between Aki6PS and SP6A1. The W14/15 esterase is related to cold tolerance of gentian winter buds, and the W14/15 alleles of Aki6PS and SP6A1 are known as 15a^′^/15a^′^ and 14b1/14b1^′^, respectively [14]. The primer sets for the functional markers are shown in Table 6. The genomic sequences of *GtFT2* and *GtTFL1* have no large insertions or deletions between Aki6PS and SP6A1. Therefore, CAPS markers were used for *GtFT2* and *GtTFL1* by digesting the amplified fragments with *Hae* III and *Sau*3A I, respectively.

PCR amplification was performed in a 20-μL reaction mixture containing 20 ng genomic DNA, 0.5 μM each primer (as shown in Table 6), 0.2 mM dNTPs, 1× *Ex* buffer, and 0.25 units *Ex Taq* polymerase. The PCR conditions were as follows: 94°C for 2 min; 30 cycles at 95°C for 20 s, 60°C for 40 s, and 72°C for 1 min; and a final extension at 72°C for 5 min. The amplified fragments were separated on the HEGS running system or 2% agarose gels in TBE buffer, and then photographed and analyzed as described above.

### Evaluation of stem pigmentation

Stem colors were investigated three times in the mapping population. Green and red stem colors were scored as *G. triflora* (A) and *G. triflora* × *G. scabra* hybrid (H) genotypes, respectively.

### Linkage analysis

We used 308 markers, including 101 SSRs, 38 REMAPs, 103 AFLPs, 54 long RAPDs, 11 functional markers, and one phenotypic trait to construct a genetic linkage map using JoinMap ver. 4.1 (Kyazma, Wageningen, Netherlands). The Kosambi function was used to convert recombination units into genetic distances. The mapping analysis was conducted by using a minimum LOD score of 9.0. Distorted markers analyzed by the chi-square test were used in the construction of the linkage maps.

## Abbreviations

AFLP: Amplified fragment length polymorphism; ANS: Anthocyanidin synthase; bHLH: Basic helix-loop-helix; CAPS: Cleaved amplified polymorphic sequence; CHS: Chalcone synthase; CHI: Chalcone isomerase; DH: Doubled haploid; F3^′^5^′^H: Flavonoid 3^′^,5^′^-hydroxylase; FHT: Flavanone 3-hydroxylase; FT: Flowering locus T; ISSR: Inter-simple sequence repeat; LTR: Long terminal repeat; MITE: Miniature inverted-repeat in transposable element; PBS: Primer binding site; QTL: Quantitative trait loci; RAPD: Random amplification polymorphic DNA; REMAP: Retrotransposon microsatellite amplified polymorphism; SCAR: Sequence characterized amplified region; SSR: Simple sequence repeat; TDR: Terminal direct repeat; TFL1: Terminal flowering 1; TRIM: Terminal repeat retrotransposon in miniature.

## Competing interests

The authors declare that they have no competing interests.

## Authors’ contributions

TN participated in the design of the experiments, and developed and genotyped the SSR, AFLP and functional markers, performed statistical analysis, data interpretation and wrote the paper. EY developed and genotyped the RAPD and REMAP markers. TH participated in the design of the experiments and produced populations for genetic analysis. MS developed and genotyped the SSR and AFLP markers. YU developed the SSR markers. MN participated in the design of the experiments and wrote the paper. All the authors read and approved the manuscript.
